# Protective Effects of Hydroxyphenyl Propionic Acids on Lipid Metabolism and Gut Microbiota in Mice Fed a High-Fat Diet

**DOI:** 10.3390/nu15041043

**Published:** 2023-02-20

**Authors:** Jingling Guo, Pan Wang, Yifan Cui, Xiaosong Hu, Fang Chen, Chen Ma

**Affiliations:** 1 Key Laboratory of Fruits and Vegetable Processing, Ministry of Agriculture, Engineering Research Centre for Fruits and Vegetables Processing, National Engineering Research Center for Fruit and Vegetable Processing, College of Food Science and Nutritional Engineering, China Agricultural University, Beijing 100083, China; 2 Beijing Key Laboratory of Agricultural Products of Fruits and Vegetables Preservation and Processing, Key Laboratory of Vegetable Postharvest Processing, Ministry of Agriculture and Rural Affairs, Institute of Agri-Food Processing and Nutrition, Beijing Academy of Agriculture and Forestry Sciences, Beijing 100097, China

**Keywords:** hydroxyphenyl propionic acid, gut microbiota, NAFLD, lipid metabolism, SCFAs

## Abstract

Gut microbiota imbalances lead to the pathogenesis of non-alcoholic fatty liver disease (NAFLD), which is primarily accompanied by hepatic steatosis. Hydroxyphenyl propionic acids (HPP) have shown great potential in inhibiting lipid accumulation but their protective effects concerning NAFLD and intestinal microbiota have remained unclear. In this paper, we investigated the efficacies of 3-HPP and 4-HPP on hepatic steatosis and gut flora in mice fed a high-fat diet (HFD). We found that 3-HPP and 4-HPP administration decreased body weight and liver index, ameliorated dyslipidemia, and alleviated hepatic steatosis. Furthermore, 3-HPP and 4-HPP enhanced the multiformity of gut microbiota; improved the relative abundance of *GCA-900066575*, *unidentified_Lachnospiraceae*, and *Lachnospiraceae_UCG-006* at genus level; increased concentration of acetic acid, propionic acid and butanoic acid in faeces; and reduced systemic endotoxin levels in NAFLD mice. Moreover, 4-HPP upregulated the relative abundance of genera *Rikenella* and downregulated the relative abundance of *Faecalibaculum*. Furthermore, 3-HPP and 4-HPP regulated lipid metabolism and ameliorated gut dysbiosis in NAFLD mice and 4-HPP was more effective than 3-HPP.

## 1. Introduction

Non-alcoholic fatty liver disease (NAFLD) is a disorder in which lipid accumulation appears in more than 5% of the liver without excessive alcohol consumption [1]. As prevalent chronic liver diseases worldwide, NAFLD harms billions of people and the disease can develop to steatohepatitis, fibrosis, cirrhosis, and hepatocellular carcinoma [2]. The hallmark of NAFLD is excessive neutral lipids such as triglyceride (TG) and cholesterol accumulation in hepatocytes [3]. The acquisition of TG and cholesterol in the liver includes biosynthesis from acetyl CoA or importation from blood; their disposal occurs through delivery to blood or conversion to other molecules [4,5]. As the liver was not served as a lipid storage organ, imbalance among these processes gradually induces hepatic steatosis and dyslipidemia [4]. However, the pathogenesis of NAFLD is intricate and several studies have suggested that gut microbiota could regulate lipid metabolism [6,7].

Gut microbiota is a community of plentiful archaea, bacteria, eumycete, and bacteriophages that coexist in the colon; dysbiosis of this community has been repeatedly observed in NAFLD [8,9]. Aron-Wisnewsky et al. summarized that the relative abundance of *Escherichia*, *Shigella*, *Ruminococcus*, and *Blautia* is increased and *Lactobacillus* is decreased at the genera level in NAFLD patients by contrast to healthy volunteers [10]. Demir et al. found that the faecal fungi composition in fibrosis patients was characterized by a higher log-ratio of Mucor sp./Saccharomyces cerevisiae [11]. In the western diet-fed mice, gradually reducing abundances of *Clostridia* and *Ruminococcaceae* followed the occurrence of NAFLD [12]. Furthermore, the connection between gut flora and NAFLD was generally explored in recent years. A high-fat diet (HFD) failed to induce dyslipidemia and lipid accumulation of liver in germ-free (GM) mice [13]. However, transplanting gut flora from HFD fed mice to GM mice reproduced phenotypes of NAFLD such as fasting hyperglycaemia, insulinaemia, and hepatic steatosis [14]. These results demonstrated that gut microbiota played a causal role in NAFLD development. Several mechanisms may interpret how gut microbiota cause the pathogenesis of NAFLD. *Desulfovibrio*, *Escherichia coli*, and other gram-negative genus in gut microbiota could release endotoxins which would enter systematic circulation through impaired gut barriers [15]. It has been demonstrated that endotoxin induced low-grade inflammation by activating Toll-like receptor 4 signal pathway and caused lipid accumulation of liver by upregulating gene expression of fatty acid synthase (*fasn*) and acetyl-coenzyme A carboxylase α (acaca) [16,17]. Besides, short chain fatty acids (SCFA), derived from gut microbiota, could inhibit lipid accumulation, and suppress inflammation [18,19]. Therefore, the maintenance of microbial homeostasis is important for protection against NAFLD.

Hydroxyphenyl propionic acids (HPPs), widespread phenolic acid, were able to alleviate inflammation and decrease lipid content of adipocytes in vitro [20,21]. They came from biotransformation of other complex polyphenol by gut microbiota [22,23,24]. It has been extensively confirmed that polyphenol can shift gut flora populations by stimulating beneficial bacteria and reducing pathogenic microbial species [25,26,27]. Nevertheless, except for parent polyphenol, the impacts of HPPs on the richness and composition of gut microbiota in NAFLD were unclear. Therefore, the aim of this study was to investigate effects of 3-HPP and 4-HPP on gut flora and related lipid metabolism in HFD induced NAFLD model. Intraperitoneal injection was selected to enhance the bioavailability of HPPs. It is demonstrated that 3-HPP and 4-HPP altered the diversity and composition of gut microbiota, decreased serum lipid contents, and ameliorated lipid aggregation of liver.

## 2. Materials and Methods

### 2.1. Materials

Both 3-HPP (C_9_H_10_O_3_, FW 166.17, purity > 98%) and 4-HPP (C_9_H_10_O_3_, FW 166.17, purity > 98%) were obtained from Aladdin (Shanghai, China). Poly (ethylene glycol) average Mn400 (PEG400) and physiological saline were purchased from Solarbio (Beijing, China). The 3-HPP and 4-HPP were dissolved in PEG and diluted with physiological saline to 24 mg/mL. Assay kits used for the detection of aspartate transaminase (AST), TG, high-density lipoprotein (HDL-c), free fatty acid (FFA), LDL-c, alanine transaminase (ALT), and total cholesterol (TC) levels were provided by Jiancheng (Nanjing, China). The enzyme-linked immunosorbent assay (ELISA) kit for detecting endotoxin was obtained from Genelab (Beijing, China). RNA easy fast kits (DP451) used for RNA isolation were obtained from TIANGEN (Beijing, China) and SYBR qPCR Master Mix was from Vazyme (Nanjing, China). D12450J and D12492 were purchased from Research Diets, Inc. (New Brunswick, NJ, USA).

### 2.2. Animal Experimental Scheme

The method of the animal experiment was adapted from a previous work with minor modifications [28]. A total of 24 C57BL/6J mice (male, a month of age) were bought from Vital River Laboratory Animal Technology Co., Ltd. (Beijing, China) and housed under the SPF environment (22 ± 2 °C, 50% ± 5 humidity) with 12 h light-dark cycle. The mice had free access to water and feed. After acclimatization for 10 days, all mice were randomly assigned to four groups (n = six per group, three mice per cage): (1) the normal diet (ND) group raised by a normal diet (D12450J), (2) the HFD group raised by an HFD (D12492), (3) the 3-HPP group raised by an HFD, (4) the 4-HPP group raised by an HFD. The dosage and frequency of 3-HPP and 4-HPP administration were 24 mg/kg body weight (BW) and twice per week, respectively. Mice received 3-HPP and 4-HPP for 13 weeks by intraperitoneal injection and PEG with saline as a control. We recorded the BW and diet intake of the mice twice per week. All experimental procedures were conducted according to the Animal Experimental Ethics Committee of China Agricultural University (reference number: AW70702202-4-1) and followed the criterion of National Research Council Guidelines.

### 2.3. Sample Collection

On the 87th day, all mice were softly stimulated in a sterile box to collect fecal samples (5–6 grains per mouse). The feces of each mouse were loaded into a sterile cryovial and stored in a −80 °C refrigerator. All mice fasted for 16 h with enough water. Then they were intraperitoneally injected with 1% sodium pentobarbital for anaesthesia. After sacrifice, we collected serum, liver, and colon of mice, which were immediately stored on dry ice and then transferred to a −80 °C refrigerator until further use.

### 2.4. Measurements of Serum Biochemicals and Liver Lipids Levels

Not only the content of TG, HDL-c, TC, FFA, LDL-c, and endotoxin, but also the activity of AST and ALT in serum were measured following instructions of test kits. TC and TG in the liver were isolated with absolute ethyl alcohol and their content was determined as described in the serum. The levels of endotoxin in the serum were measured based on the ELISA assay kit instructions.

### 2.5. Histopathological Examination

The histopathological procedure was performed as a previous study with slight modifications [29]. The fresh liver was fixed with 4% paraformaldehyde and sliced. After fixation, the liver sample was dehydrated with 15% (*w*/*v*) and 30% (*w*/*v*) sucrose solution successively at 4 °C. Then the liver sample was embedded by an optimal cutting temperature compound, sectioned (6–7 μm) and stained with hematoxylin-eosin (H&E) or oil red o (ORO). The slices were then photographed using a microscope (Nikon, Tokyo, Japan) at 200× magnification.

### 2.6. RNA Extraction and Quantitative Real-Time Polymerase Chain Reaction (qRT-PCR)

The procedure for qRT-PCR followed a previous study with few modifications [30]. Total RNA in liver samples were extracted by following assay kit instructions and then reversely transcribed to cDNA. The reaction system of qRT-PCR contained 2 μL cDNA, 0.7 μL forward primer, 0.7 μL reverse primer, 6.6 μL ddH2O, and 10 μL SYBR qPCR Master Mix. The PCR program was run on the BioRad CFX96 System, which included a first step at 95 °C for 5 min, a second step for 40 PCR cycles of 95 °C for 10 S, 60 °C for 30 S, 72 °C for 15 S, and a last step for melting curve. The primers of targeted genes are shown in Table 1 and the results of qRT-PCR were normalized to the *gapdh* expression and calculated by the 2^−∆∆Ct^ method.

### 2.7. Gut Microbiota Analysis by 16S rRNA Sequencing

Procedures of 16S rRNA sequencing and analysis were developed from a published study with some modifications [31]. The DNA in mice feces was extracted by TIANamp Stool DNA Kit (TIANGEN, Beijing, China). After isolation, the quality and concentration of DNA were detected using Nanodrop 2000 (Thermo Fisher, Darmstadt, Germany) and 0.75% agarose gel electrophoresis (AGE). The V3-V4 regions of bacterial 16S rDNA were amplified by barcode primers (341F: CCTAYGGGRBGCASCAG; 806R: GGACTACNNGGGTATCTAAT) and DNA polymerase (NEB, Massachusetts, USA). Then, the 2% AGE was used to examine the quality of 16S rDNA amplification and recycled using a DNA Gel Extraction Kit with Magnetic Beads (Beyotime, Nanjing, China). The library was constructed according to the instructions of the TruSeq^®^ DNA PCR-Free Sample Preparation Kit (Illumina, San Diego, CA, USA) and amplicon sequencing was performed on the NovaSeq 6000 platform.

### 2.8. Measurement of Short Chain Fatty Acids (SCFAs) Content in Feces

Analysis concentrations of SCFA was performed with reference to the method of Hsu et al. with slight modifications [32]. About 20 mg of mice feces were mixed with 800 μL 0.5% phosphoric acid solution which contained 2-ethyl butyric acid (internal standard) at the concentration of 10 μg/mL. After ultrasound and centrifugation, the supernatant was extracted with n-butanol. The qualitative and quantitative analysis of SCFA was accomplished using a GC/MSD system with an Agilent HP-FFAP capillary column (30 m × 0.25 mm × 0.25 µm) (Agilent Technologies Inc., Palo Alto, CA, USA). A mix of propionic acid, acetic acid, butyric acid, isobutyric acid, valeric acid, isovaleric acid, hexanoic acid, and isohexanoic acid (25 μg/mL each, Sigma, Saint Louis, MO, USA) was used for calibration. We calculated the concentration of these SCFA based on standard solutions corrected by internal standard and the integral area of sample curves.

### 2.9. Statistical Analysis

All data were expressed as means ± standard deviations (SDs). The difference between the four groups was analysed by one-way analysis of variance using SPSS 20.0. Duncan’s test was used for the comparison between groups. *p* < 0.05 was deemed significant for all tests. Non-metric multidimensional scaling (NMDS) was used to analyse discrepancies among samples. The key genera between the HFD and HPP (or ND) groups were analysed by the Wilcoxon rank sum test at *p* < 0.05. Phylogenetic Investigation of Communities by Reconstruction of Unobserved States 2 (PICRUSt2) and Kyoto Encyclopedia of Genes and Genomes (KEGG) databases were selected to predict the metabolic function of gut microbiota.

## 3. Results

### 3.1. HPPs Reduced BW and Liver Index in HFD Mice

**Figure 1:**
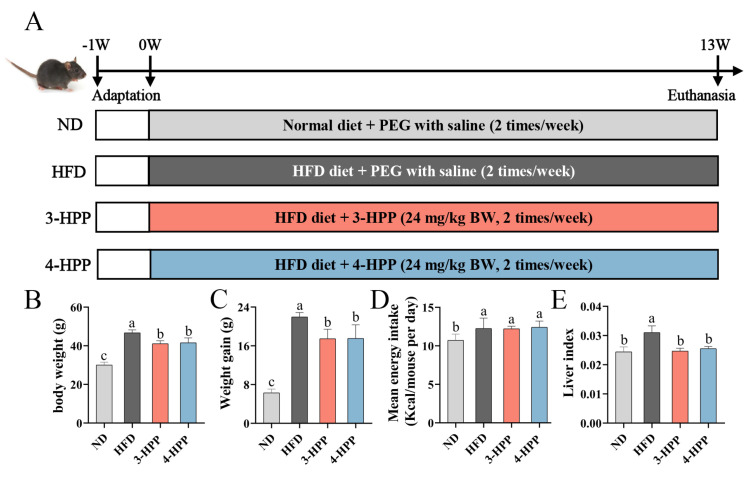
The influence of 3-hydroxyphenyl propionic acid (3−HPP) and 4−HPP on physiological indexes. (**A**) Schematic diagram of animal experiment period. (**B**) Final body weight (BW). (**C**) BW gain. (**D**) Energy intake. (**E**) Liver index. Data are expressed as mean ± SD. n = 6 if not specified. According to Duncan’s test, mean values with different letters mean significant differences at *p* < 0.05.

To explore the influence of 3-HPP and 4-HPP on NAFLD, C57BL/6J mice were intraperitoneally administrated with 3-HPP and 4-HPP and continuously fed an HFD for 13 weeks (Figure 1A). The BW of mice in four groups was without a marked difference in the initial experimental period. After being raised by HFD for 13 weeks, the BW, BW gain, and liver index of HFD mice were significantly increased compared to ND mice (Figure 1B–D). The 3-HPP and 4-HPP treatments attenuated these characteristics without affecting energy intake, suggesting that the efficacy of 3-HPP and 4-HPP did not depend on decreased food intake (Figure 1E).

### 3.2. HPPs Improved Hepatic Steatosis and Ameliorated Liver Injury

**Figure 2:**
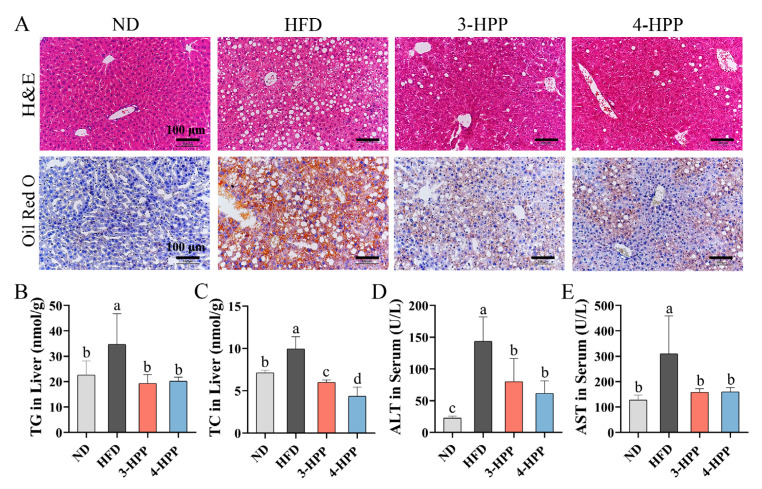
The impacts of 3-HPP and 4-HPP on hepatic steatosis and liver injury. (**A**) Graphs of H&E staining and ORO staining. (**B**) Hepatic TG levels. (**C**) Hepatic TC levels. (**D**) Serum contents of ALT. (**E**) Serum contents of AST. Data are shown as the mean ± SD. n = 6 if not specified. According to Duncan’s test, mean values with different letters mean significant differences at *p* < 0.05.

As the major organ involved in NAFLD, the histologic feature of the liver was examined by H&E and ORO staining. H&E staining revealed that an HFD-induced ballooning degeneration and large areas of lipid droplet vacuole were observed in HFD mice livers. Similarly, the stained ORO sections of HFD mice livers showed plenty of red lipid droplets, which illustrated lipid aggregation. However, areas of lipid droplet vacuole and red lipid droplet were reduced, and ballooning degeneration disappeared in histologic results following 3-HPP and 4-HPP interventions (Figure 2A). We then used test kits to detect liver lipid levels and found that hepatic TG and TC contents in HFD mice were obviously more than those in ND mice; this was decreased by 3-HPP and 4-HPP (Figure 2B,C). These results demonstrated that HPPs protected HFD mice from hepatic steatosis and hepatic lipid accumulation.

Generally, ALT and AST contents in serum are deemed to be indicators of liver damage [33]. The serum contents of AST and ALT were sharply increased after HFD feeding for 13 weeks and ameliorated by 3-HPP and 4-HPP administration (Figure 2D,E). This indicates that HPPs can protect mice from hepatic injury.

**Figure 3:**
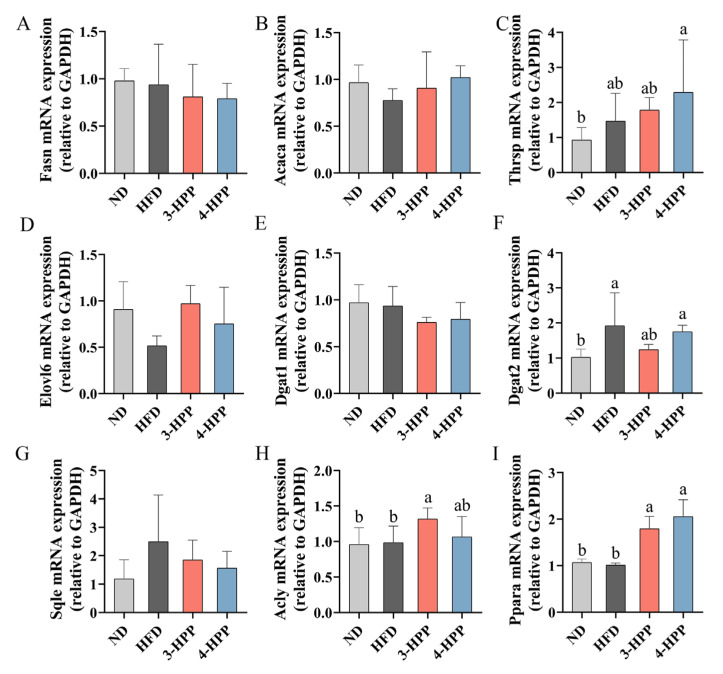
The impacts of 3−HPP and 4−HPP on gene expression of hepatic lipid metabolism. mRNA expression of (**A**) Fasn, (**B**) Acaca, (**C**) Thrsp, (**D**) Elovl6, (**E**) Dgat1, (**F**) Dgat2, (**G**) Sqle, (**H**) Acly, and (**I**) Ppara in liver. Data are shown as the mean ± SD. n = 6 if not specified. According to Duncan’s test, mean values with different letters mean significant differences at *p* < 0.05.

Then we examined hepatic gene expression of de novo lipogenesis (DNL) (Figure 3A–D), including *fasn*, *acaca*, thyroid hormone responsive (*thrsp*), and elovl fatty acid elongase 6 (*elovl6*). Except for DNL, we also explored hepatic gene expression of TG and TC synthesis, including diacylglycerol O-acyltransferase 1 (*dgat1*), diacylglycerol O-acyltransferase 2 (*dgat2*), squalene epoxidase (*sqle*), and ATP citrate lyase (*acly*) (Figure 3E–H). Gene expression of *thrsp* and *dgat2* was improved in HFD mice while 3-HPP and 4-HPP failed to reverse their upward tendency. However, 3-HPP and 4-HPP enhanced gene expression of peroxisome proliferator activated receptor alpha (*ppara*), which acts upstream of lipid metabolism (Figure 3I).

### 3.3. HPPs Affected the Maker Levels in Serum

Clinical studies have found that the majority of NAFLD patients suffer from dyslipidemia [34]. Similarly, levels of lipids such as FFA, TG, and TC and lipoproteins (LDL-c and HDL-c) in HFD mice serum were significantly increased compared to ND mice (Table 2). Nevertheless, serum levels of TG, FFA, and TC were reduced following 3-HPP and 4-HPP treatment. The 4-HPP treatment also decreased serum levels of LDL-c. These results confirm the protective efficacy of 3-HPP and 4-HPP for dyslipidemia.

Increased endotoxin levels in serum derived from intestinal microbiota have been considered a risk factor for NAFLD [35]. Long-term HFD feeding induced a marked increment of serum endotoxin in HFD mice, which was decreased by 3-HPP and 4-HPP treatment (Table 2).

### 3.4. HPPs Influenced the α and β Diversity of Gut Microbiota

**Figure 4:**
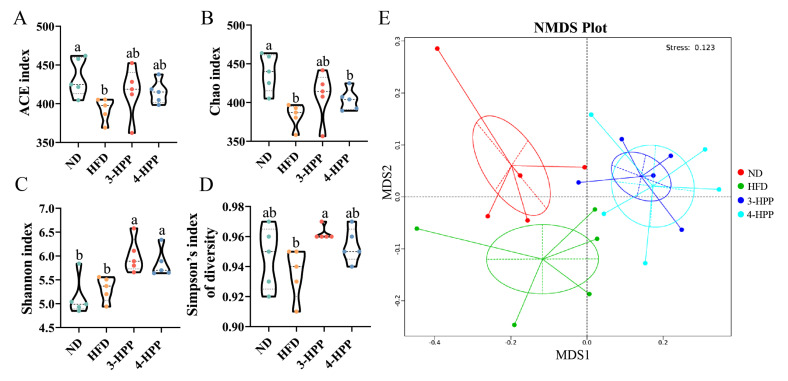
The impacts of 3−HPP and 4−HPP on α and β diversity of gut flora. ACE (**A**), Chao (**B**), and Shannon (**C**) indexes. (**D**) Simpson’s index of diversity ACE. (**E**) NMDS analysis based on Weighted Unifrac distance. Data are expressed as mean ± SD. n = 5 if not specified. According to Duncan’s test, mean values with different letters denote significant differences at *p* < 0.05.

Recent evidence indicates that gut flora is related to the pathogenesis of NAFLD [36]. Therefore, the α and β diversity of gut flora was investigated in this study. HFD feeding significantly decreased ACE and Chao indexes in comparison with ND feeding, which inflected the richness and abundance of gut microbiota. The Simpson’s index of diversity was reduced in HFD mice, illustrating that an HFD decreased gut microbiota diversity. HPPs significantly increased the Shannon and Simpson’s indexes of diversity and had an upload tendency for the ACE and Chao indexes (Figure 4A–D). Additionally, the results of NMDS analysis illustrated that confidence circles among the ND, HFD, and HPP groups were not overlapping, illustrating differences among the gut microbiota composition of these groups (Figure 4E). This result suggests that 3-HPP and 4-HPP administration affected the diversity and composition of gut flora.

### 3.5. HPPs Modulated the Composition of Gut Flora

**Figure 5:**
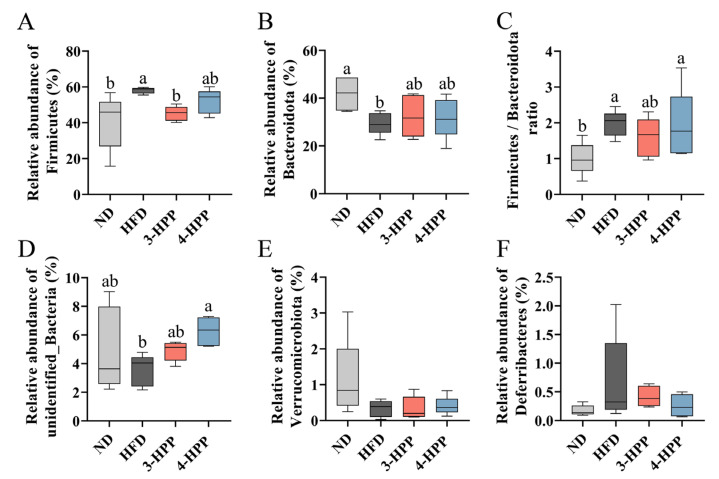
The effects of 3−HPP and 4−HPP on the relative abundance of gut microbiota at the phylum level. Relative abundance of *Firmicutes* (**A**), *Bacteroidetes* (**B**), *unidentified_Bacteria* (**D**), *Verrucomicrobita* (**E**), and *Deferribacteres* (**F**). (**C**) The ratio of *Firmicutes* to *Bacteroidetes*. Data are expressed as means ± SDs. n = 5 if not specified. According to Duncan’s test, mean values with different letters denote significant differences at *p* < 0.05.

The results of taxonomic analysis at phylum and genus levels further displayed gut microbiota composition changes (Supplementary Figure S1). The relative abundance of five main phyla is shown in Figure 5. Among the four groups of mice, phylum *Firmicutes* (50.29%) and *Bacteroidetes* (32.25%) were most widespread, followed by *unidentified_Bacteria* (6.16%), *Verrucomicrobia* (2.17%) and *Deferribacteres* (1.5%). HFD feeding not only increased the relative abundance of *Firmicutes* and the ratio of *Firmicutes* to *Bacteroidetes* (F/B) but also decreased the relative abundance of *Bacteroidetes* and *unidentified_Bacteria*, which was partially reversed by 3-HPP and 4-HPP administration (Figure 5A–D). These treatments did not affect the relative abundance of *Deferribacteres* or *Verrucomicrobia* (Figure 5E,F).

**Figure 6:**
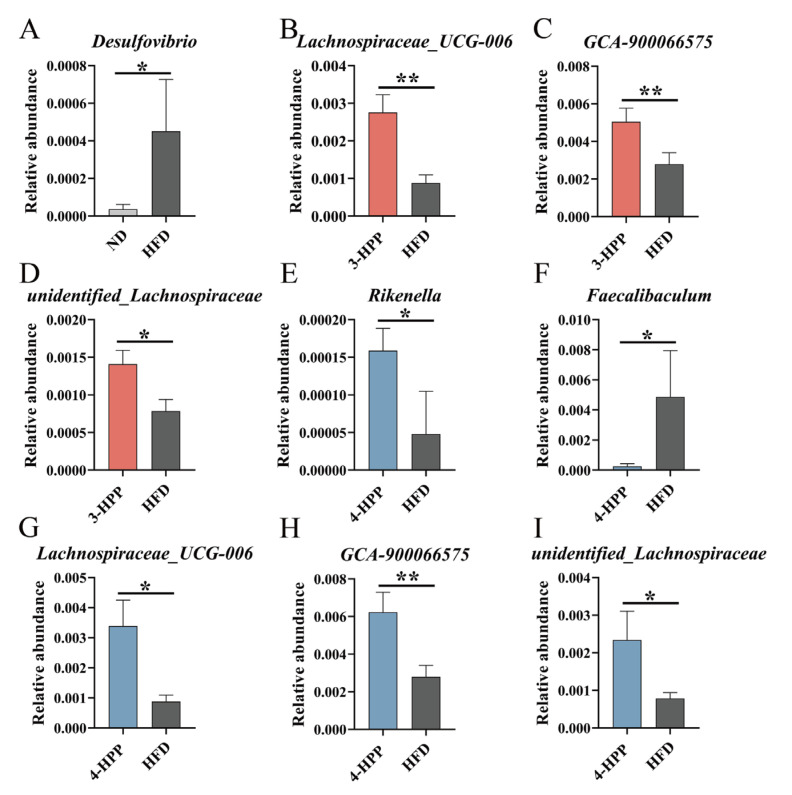
The effects of 3−HPP and 4−HPP on the relative abundance of key genera in different groups. Relative abundance of *Desulfovibrio* (**A**) between the ND and HFD groups. Relative abundance of *Lachnospiraceae_UCG-006* (**B**), *GCA-900066575* (**C**), and *unidentified_Lachnospiraceae* (**D**) between the 3−HPP and HFD groups. Relative abundance of *Rikenella* (**E**), *Faecalibaculum* (**F**), *Lachnospiraceae_UCG-006* (**G**), *GCA-900066575* (**H**), and *unidentified_Lachnospiraceae* (**I**) between the 4−HPP and HFD groups. Data are expressed as means ± SDs. n = 5 if not specified. The results were analysed by a Wilcoxon rank sum test. * *p* < 0.05 and ** *p* < 0.01 compared to the HFD group.

The relative abundance of the top 10 genera accounted for 43% of the total; the top 10 genera were *Blautia*, *Rikenellaceae_RC9_gut_group*, *Dubosiella, Bacteroides*, *Akkermansia*, *Ruminococcus*, *Lactobacillus*, *Ligilactobacillus* and *Alistipes* (Supplementary Figure S1B). The key genera between the HFD and HPP groups were selected according to a Wilcoxon rank sum test. HFD feeding significantly increased the relative abundance of *Desulfovibrio*, which is related to inflammation (Figure 6A). With 3-HPP and 4-HPP treatment, the relative abundance of *GCA-900066575*, *unidentified_Lachnospiraceae*, and *Lachnospiraceae_UCG-006* increased, which belong to *Lachnospiraceae* family (Figure 6B–D,G–I). 

### 3.6. HPPs Affected the Predicted Functions and SCFAs Production of Gut Flora

**Figure 7:**
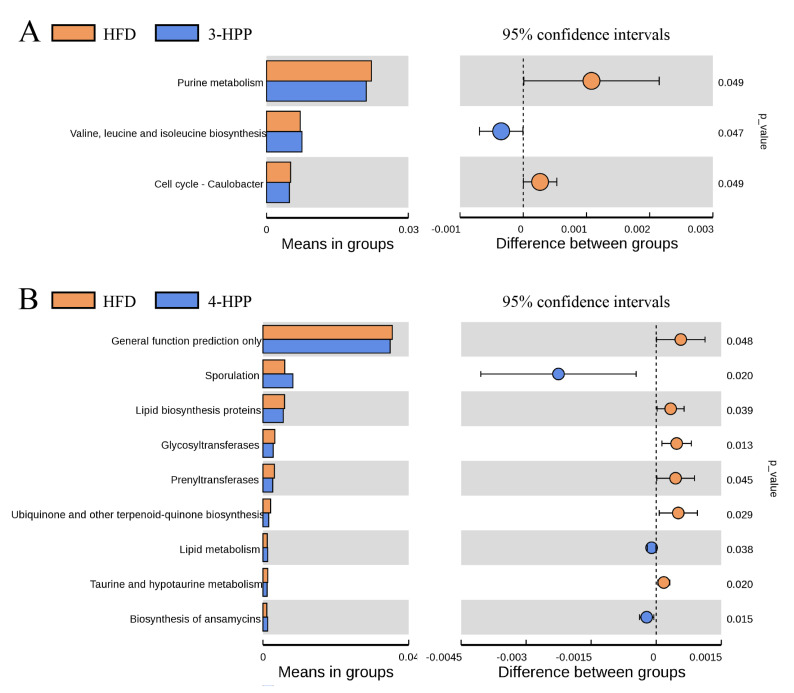
The effects of 3−HPP and 4−HPP on the predicated metabolic profile of gut microbiota. (**A**) Comparison between HFD and 3−HPP. (**B**) Comparison between HFD and 4−HPP. The key metabolic pathways predicted by PICRUSt2 and KEGG were selected based on a t-test at *p* < 0.05. n = 5 if not specified. The colour of the circle is the same as the group with a higher function. The line segment indicates the 95% confidence interval.

OTUs from 16S rRNA sequencing were selected to predict the distinction in metabolic function between HFD and HPP groups by combining the PICRUSt2 and KEGG databases. HFD feeding upregulated nitrogen metabolism and enriched amino acid metabolism in comparison with ND feeding (Supplementary Figure S2). By contrast to HFD group, the 3-HPP group markedly increased valine, leucine and isoleucine biosynthesis (Figure 7A). The administration of 4-HPP inhibited lipid biosynthesis and glycosyltransferases and increased lipid metabolism, which is useful for explaining the beneficial efficacy of 4-HPP for hepatic and serum fat contents (Figure 7B).

**Figure 8:**
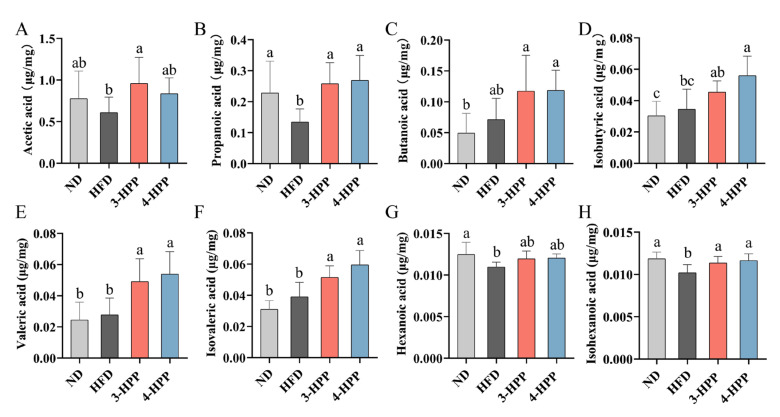
The effects of 3−HPP and 4−HPP on SCFAs content of feces. Fecal concentrations of acetic acid (**A**), propanoic acid (**B**), butanoic acid (**C**), isobutyric acid (**D**), valeric acid (**E**), isovaleric acid (**F**), hexanoic acid (**G**), and isohexanoic acid (**H**). n = 6 if not specified. According to Duncan’s test, mean values with different letters mean significant differences at *p* < 0.05.

In addition to the disruption of metabolic function, the content of acetic acid, propanoic acid, hexanoic acid, and isohexanoic acid in HFD mice was distinctly reduced when compared to ND mice. Nevertheless, 3-HPP and 4-HPP obviously enriched concentrations of most SCFAs except for isohexanoic acid (Figure 8A–H). These data suggested that HPPs could effectively regulate the content of SCFAs.

## 4. Discussion

NAFLD is becoming an increasingly serious public health problem, with high prevalence among both children and adults [37]. Long-term HFD-induced mice models have been widely used to investigate NAFLD development. In this study, HFD feeding for 13 weeks increased BW and liver index and induced dyslipidemia, which is parallel to NAFLD development in humans and other models [38,39]. The use of 3-HPP and 4-HPP supplementation not only significantly reduced liver index and BW but also lessened the contents of TG, LDL-c, and TC in serum. We found 4-HPP was more effective at reducing lipid levels in the liver and serum. These efficacies of HPPs were similar to those of other phenolic acids such as caffeic and chlorogenic acid [40,41]. Although HPPs had few impacts on gene expression of lipogenesis, they improved mRNA expression of PPARα. PPARα is a nuclear receptor with pivotal regulation functions in fatty acid oxidation, transport, and ketogenesis [42]. Chi et al. found that zinc complex of ulvan oligosaccharide enhanced fatty acid oxidation in the liver via the metal regulatory transcription factor 1/PPARα pathway [43]. As fatty acid is the main material for TG synthesis, HPPs might alleviate hypertriglyceridemia by activating hepatic PPARα gene expression to reduce fatty acid levels.

The liver is the major organ for lipid metabolism and excessive hepatic lipid accumulation can induce NAFLD. Liver biopsies of NAFLD patients show pathological changes such as lipid vacuole, lobular inflammation and hepatocellular ballooning [44]. Increments in ALT and AST levels in serum are associated with NAFLD and liver damage [33]. Interestingly, we found that 3-HPP and 4-HPP prevented pathological changes in the livers of HFD mice and increased serum ALT and AST levels. These results suggest that HPPs treatment ameliorates hepatic steatosis and liver injury. Accordingly, Cui et al. found that 3-HPP from dietary acteoside could ameliorate oxidative stress, decreasing lipid peroxidation, and reducing inflammation in the acute liver injury mice model, which demonstrated the hepatoprotective effect of 3-HPP [21]. Based on beneficial impacts of HPPs on NAFLD, their mechanism of improving NAFLD was further investigated by 16S rRNA sequencing.

Gut microbiota played critical roles in host metabolism and health. Recent evidence indicates that gut microbiota is related to NAFLD and could be a targeted therapy [9,45]. Shen found that the diversity and abundance of gut flora are lower in NAFLD patients compared to healthy subjects [46]. In our study, an HFD reduced the diversity and richness of gut microbiota, and this was reversed by 3-HPP and 4-HPP treatment. The results of NMDS showed that HFD feeding and HPPs supplement influenced the composition and structure of intestinal microbiota, indicating that HPPs can modulate gut microbiota. In this study, HFD feeding upregulated the relative abundance of Firmicutes and downregulated the relative abundance of Bacteroidetes, which caused the F/B ratio to increase. These changes in phylum have also been observed in other NAFLD models and obese humans [36,47]. HPPs administration tended to reduce the ratio of F/B. The relative abundance of *Dubosiella* genera was lower and the relative abundance of *Desulfovibrio* genera was higher in HFD mice compared to ND mice. *Dubosiella*, part of the *Erysipelotrichaceae* family, has a negative correlation with the gene expression of tumor necrosis factor-α and interleukin-1β [48]. *Desulfovibrio*, a kind of sulphate-reducing bacteria, is enriched in gastric cancer patients and its metabolite (hydrogen sulphide) induces the production of NO and IL-1β to promote inflammation [49]. The change in the relative abundance of *Dubosiella* and *Desulfovibrio* in HFD mice reflected gut dysbiosis during NAFLD. 3-HPP and 4-HPP administration improved the relative abundance of genera such as *GCA-900066575*, *unidentified_Lachnospiraceae*, and *Lachnospiraceae_UCG-006*, part of the *Lachnospiraceae* family. With 4-HPP treatment, the relative abundance of *Rikenella* genera and *Faecalibaculum* genera increased and decreased, respectively. The *Lachnospiraceae* family and *Rikenella* genera are the producers of SCFA [50,51]. *Faecalibaculum*, a proinflammatory genus, can induce depression-like phenotypes [52,53]. The regulation of 3-HPP and 4-HPP concerning the structure and composition of gut microbiota partly explained their amelioration of gut dysbiosis, and 4-HPP modulated gut microbiota composition to a stronger extent than 3-HPP. Cueva et al. discovered that 4-HPP was more effective than 3-HPP in inhibiting the growth of *Lactobacilli*, *E. coli*, and *Staphylococcus aureus* strains, which could explain the better impacts of 4-HPP on gut microbiota [54].

The regulation of gut microbiota on host metabolism majorly depended on its metabolic functions and metabolites, except for relying on its composition and abundance. SCFAs, such as propionate, butyrate, and acetate, are microbial products from carbohydrate fermentation. After utilization of enterocytes, SCFAs could enter the liver by portal vein transport to regulate hepatic lipid metabolism [55]. den Besten et al. discovered that sodium of propionate, acetate, and butyrate shifted hepatic lipogenesis to lipid oxidation by activating AMP-activated protein kinase/PPARγ signal pathway [56]. In our results, HPPs enhanced colonic production of acetic acid, propanoic acid, and other SCFAs, which partly explained the lipid-lowering effect of HPPs.

Endotoxins come from gram-negative bacteria and are a causative agent of NAFLD [35]. Wu et al. found that an HFD increased endotoxin levels in the serum and livers of NAFLD mice [57]. In our study, long-term HFD feeding also increased endotoxin levels in serum and this was reversed by 3-HPP and 4-HPP administration. In addition, HPPs enriched fecal content of butanoic acid. It has been suggested that butyrate could ameliorate gut barriers impairment by upregulating the expression of zonula occluden-1 and decrease inflammation by promoting generation of interleukin-22 through regulating histone deacetylase and G-protein receptor 41 [58,59]. Therefore, the systematic inflammation induced by endotoxin might be ameliorated by enrichment of butanoic acid through HPPs. The mechanism by which 3-HPP and 4-HPP reduce endotoxin content remains unclear and requires further investigation.

## 5. Conclusions

In conclusion, 3-HPP and 4-HPP significantly ameliorated the degree of NAFLD in mice models by reducing liver and serum lipid contents, thereby alleviating hepatic steatosis, inhibiting liver injury, and reducing endotoxin levels in serum. The results of high-throughput 16S rRNA gene sequencing showed that HPPs administration enhanced the diversity of gut microbiota and had a tendency to reduce the ratio of F/B. We found that 4-HPP treatment enriched *Rikenella* genera and reduced the relative abundance of *Faecalibaculum.* Furthermore, 4-HPP was more active than 3-HPP in decreasing lipid content and modulating gut microbiota. The causality between gut microbiota and the protective effects of HPPs for NAFLD is an interesting topic for future research. Our findings provide a valuable foundation for NAFLD prevention by HPPs.

## Figures and Tables

**Table 1:** Primers of qRT-PCR.

Gene	Forward Primer (5′ to 3′)	Reverse Primer (5′ to 3′)
*fasn*	ATGAGCGCACCTTTGATGAC	GATGCCGTCAGGTTTCAGTC
*acaca*	ATGTCTGGCTTGCACCTAGT	ATCGCATGCATTTCACTGCT
*thrsp*	ACCTAGAAGCCCAGTTCCAC	CTACAGAACCTGCCCTGTCA
*elovl6*	GTGCAGAGGCTTGAGAAGTG	TAATCTCCGCAGGCCCTTAG
*dgat1*	GTGCCATCGTCTGCAAGATT	GATCAGCATCACCACACACC
*dgat2*	CTTCTCTGTCACCTGGCTCA	CGTGTTCCAGTCAAATGCCA
*sqle*	TCGCTGCCTTCTCGGATATT	CTGAGGTAGCTGCTCCTGTT
*acly*	GCCAAGACCATCCTCTCACT	GAAGTTTGCAATGCTGCCT
*ppara*	TACTGCCGTTTTCACAAGTGC	AGGTCGTGTTCACAGGTAAGA
*gapdh*	AACGGATTTGGCCGTATTGG	CATTCTCGGCCTTGACTGTG

**Table 2:** The influence of 3−HPP and 4−HPP on serum biochemical levels.

Parameter	ND	HFD	3-HPP	4-HPP
TC (mg/dL)	2.07 ± 0.32 ^c^	6.26 ± 0.13 ^a^	5.31 ± 0.12 ^b^	4.99 ± 0.18 ^b^
TG (mg/dL)	0.81 ± 0.19 ^c^	1.37 ± 0.07 ^a^	1.02 ± 0.05 ^b^	0.99 ± 0.03 ^b^
HDL-c (mmol/L)	1.25 ± 0.39 ^b^	4.06 ± 0.08 ^a^	3.59 ± 0.06 ^a^	3.59 ± 0.05 ^a^
LDL-c (mmol/L)	0.42 ± 0.06 ^c^	1.18 ± 0.24 ^a^	1.04 ± 0.36 ^a,b^	0.92 ± 0.07 ^b^
Endotoxin (ng/L)	179.28 ± 5.28 ^c^	359.61 ± 8.29 ^a^	240.05 ± 13.66 ^b^	236.83 ± 14.51 ^b^
FFA (mmol/L)	1.27 ± 0.20 ^a,b^	1.44 ± 0.07 ^a^	1.17 ± 0.14 ^b^	1.12 ± 0.18 ^b^

Data are expressed as mean ± SD. n = 6 if not specified. According to Duncan’s test, mean values with different letters mean significant differences at *p* < 0.05.

## Data Availability

Data are available from the corresponding author on reasonable request.

## Supplementary Materials

The following supporting information can be downloaded at https://www.mdpi.com/article/10.3390/nu15041043/s1. Figure S1: The relative abundance of microbiota in ND, HFD, 3-HPP and 4-HPP groups; Figure S2: A comparison between the ND and HFD groups of the predicated metabolic profiles of gut microbiota.
