# Supplementary material for: Protective Effects of Hydroxyphenyl Propionic Acids on Lipid Metabolism and Gut Microbiota in Mice Fed a High-Fat Diet
Source: Nutrients. 2023 Feb 20;15(4):1043. doi: 10.3390/nu15041043 (PMC9959022; doi:10.3390/nu15041043)

Supplemental Figure legends

Supplementary Figure S1. The relative abundance of microbiota in ND, HFD, 3-HPP and 4-HPP groups. (A) phylum level; (B) genus level.

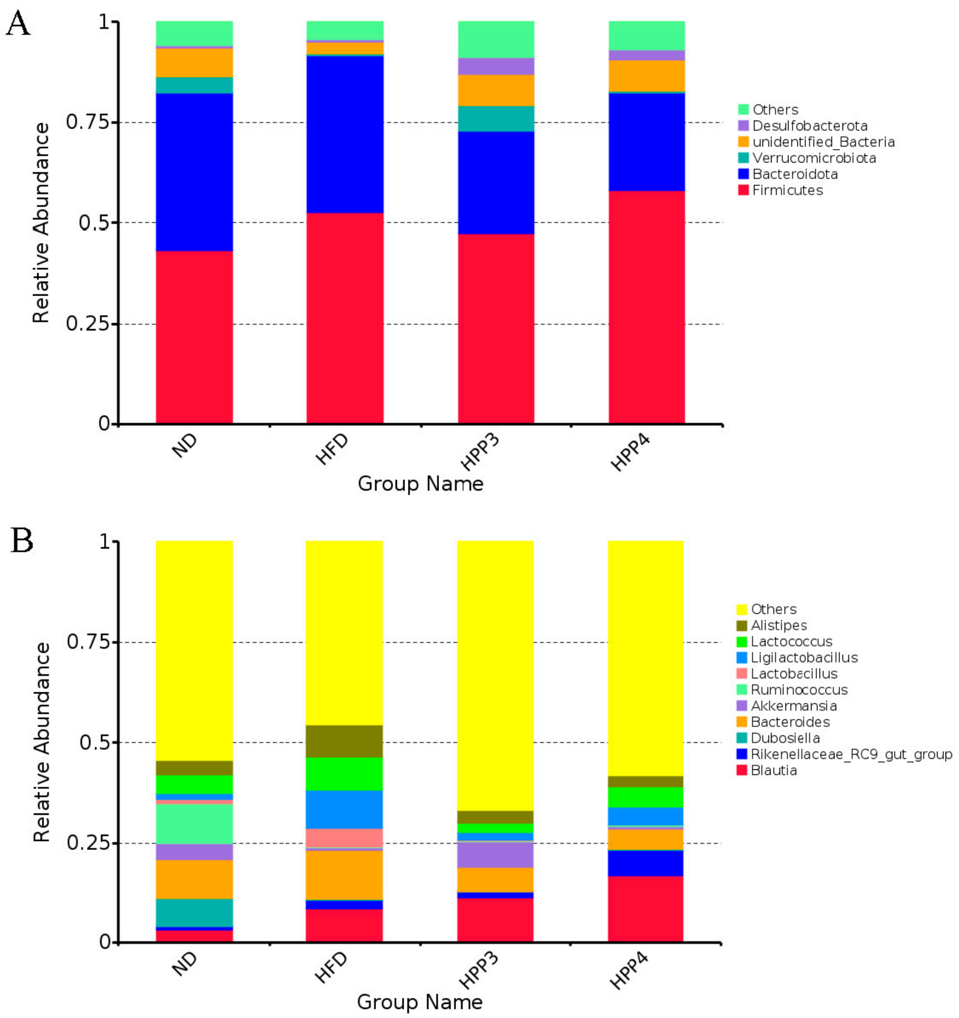

Supplementary Figure S2. A comparison between the ND and HFD groups of the predicated metabolic profiles of gut microbiota.

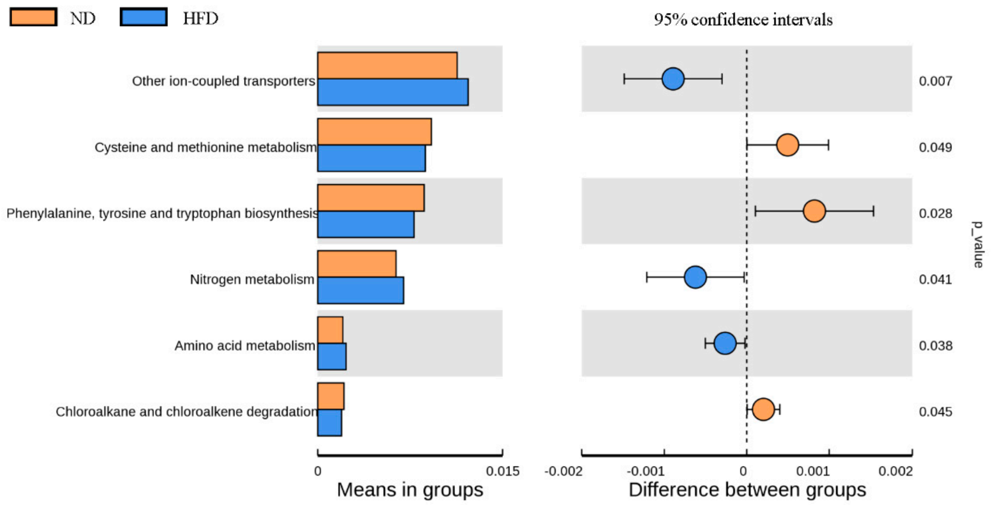

Supplement: Supplementary file 1 [file nutrients-15-01043-s001.zip › nutrients-2125200-supplementary.pdf]
